# Characterization and Antimicrobial Resistance of Non-Typhoidal *Salmonella* from Poultry Carcass Rinsates in Selected Abattoirs of KwaZulu Natal, South Africa

**DOI:** 10.3390/microorganisms13081786

**Published:** 2025-07-31

**Authors:** Bongi Beatrice Mankonkwana, Evelyn Madoroba, Kudakwashe Magwedere, Patrick Butaye

**Affiliations:** 1 Department of Biochemistry and Microbiology, University of Zululand, KwaDlangezwa, Empangeni 3886, South Africa; 201732875@stu.unizulu.ac.za; 2 Center on Emerging Infectious Diseases, Boston University, 111 Cummington Mall, Suite 140, Boston, MA 02215, USA; gwedas@yahoo.co.uk; 3 Department of Pathobiology, Pharmacology and Zoological Medicine, Faculty of Veterinary Medicine, Ghent University, Salisburylaan 133, 9820 Merelbeke, Belgium; pabutaye@cityu.edu.hk; 4 Department of Infectious Diseases and Public Health, Jockey Club College of Veterinary Medicine and Life Sciences, City University of Hong Kong, Hong Kong, China

**Keywords:** *Salmonella* Typhimurium, *Salmonella* Enteritidis, virulence, antimicrobial resistance, chicken carcass rinsates

## Abstract

Contaminated poultry is one of the major sources of food-borne non-typhoidal *Salmonella* (NTS). The aim of this study was to evaluate the presence of *Salmonella* along the slaughter process in low- and high-throughput poultry abattoirs in South Africa and to determine their characteristics. Samples were collected from 500 chicken carcass rinsates at various processing stages in three abattoirs. *Salmonella* detection and identification was conducted in accordance with the ISO 6579 methodology. NTS serotyping was performed with serotype-specific PCRs. The Kirby–Bauer disk diffusion method was used to determine antimicrobial resistance in *Salmonella.* PCR was used to analyze thirteen antimicrobial genes and four virulence genes. *Salmonella* spp. was detected in 11.8% (59/500; CI: 9.5–15) of the samples tested. The predominant serovars were *Salmonella* Enteritidis (n = 21/59; 35.59%) and *Salmonella* Typhimurium (n = 35; 59.32%). Almost all *Salmonella* isolates were susceptible to all tested antimicrobials except three. Despite the low resistance to tetracyclines at the phenotypic level, approximately half of the strains carried *tetA* genes, which may be due to “silent” antimicrobial resistance genes. Diverse virulence genes were detected among the confirmed NTS serotypes. We found a predominance of *S.* Enteritidis and *S.* Typhimurium from chicken carcasses with diverse virulence and resistance genes. As we detected differences between the slaughterhouses, an in-depth study should be performed on the risk of *Salmonella* in low- and high-throughput abattoirs. The integrated monitoring and surveillance of NTS in poultry is warranted in South Africa to aid in the design of mitigation strategies.

## 1. Introduction

The *Salmonella* genus is divided into two species: *Salmonella bongori* and *Salmonella enterica*. *Salmonella enterica* is further categorized into six subspecies: S. *enterica*, *S*. *salamae*, *S. arizonae*, *S. diarizonae*, *S. indica*, and *S. houtenae* [1]. Approximately 99% of *Salmonella* human infections are caused by a small number of serotypes from the *Salmonella enterica* subspecies *enterica* group, even though over 2610 *Salmonella enterica* serotypes have been identified [2,3]. According to the World Health Organization, as well as preliminary analysis from The Institute for Health Metrics and Evaluation (IHME), diarrheal illnesses due to NTS accounted for approximately 73.9 million cases, resulting in 61,600 fatalities in 2019 [4]. Although African countries have fewer reported instances of NTS gastroenteritis than other regions, probably due to underreporting, the frequency of invasive non-typhoidal *Salmonella* (iNTS) remains high [5]. Especially in Sub-Saharan Africa, NTS causes invasive salmonellosis and bacteraemia [6,7,8]. In Africa, the most common serovars are *Salmonella* Enteritidis and *Salmonella* Typhimurium, accounting for 26% and 25% of all *Salmonella* infections, respectively [9,10]. From 2013 to 2015, six of seven *Salmonella enterica* Enteritidis outbreaks in South Africa were foodborne [10].

It was shown before that animal-derived food products are the primary carriers of NTS [11,12]. Poultry meat accounts for most of the foodborne salmonellosis cases [13,14,15]. *Salmonella* is found in chicken gastrointestinal tracts, as well as the oviducts, and may contaminate the carcass during slaughter [16,17]. In modern abattoirs, the rapid line speed during processing keeps the birds in close proximity, which increases the potential of microbial cross-contamination between infected and uninfected carcasses [18]. In South Africa, the Meat Safety Act of 2000 (Act No. 40 of 2000) regulates the hygiene and quality of meat, as well as the quantity of units that can be slaughtered [19]. The Foodstuffs, Cosmetics, and Disinfectants Act of 1972 (Act No. 54 of 1972) further regulates microbiological food monitoring [20].

Antibiotics are used to treat *Salmonella* infections. However, in mild infections, antibiotic therapy is not recommended as salmonellosis is a self-limiting disease [21]. There are numerous reports regarding antimicrobial resistance among *Salmonella* [22,23,24,25,26,27,28,29]. Drivers of antimicrobial resistance in *Salmonella* include horizontal gene transfer, environmental factors, and the use of antibiotics in human and veterinary medicine [30]. Generally, bacteria resist antimicrobial agents through the enzymatic inactivation of antimicrobials, the modification of target proteins receptors, and extrusion by the efflux pump [31,32,33,34]. Third-generation cephalosporin resistance is of major public health importance, which is generally the result of β-lactam ring disruption by enzymes encoded by mainly a multitude of variants of *bla_CMY_*, *bla_TEM_*, *bla_SHV_*, *bla_PSE_*, *bla_OXA_*, and *bla_CTX-M_* genes [35]. Another major antimicrobial resistance of public health importance is fluroquinolone resistance, which is mediated by point mutations in the *Salmonella gyr*A and/or *parC* genes [1,36]. Taking into consideration that the infection in humans originates mainly from food animals or foods contaminated by products of food animals, antimicrobial resistance in *Salmonella* from food animals is a major public health issue [37,38]. The resistance genes can be found on plasmids, gene cassettes in integrons, or other mobile genetic elements (MGE) [1,39]. MGEs can be transferred between serovars and to other bacterial genera through horizontal gene transfer, resulting in the spread of resistance [40,41].

The main virulence factors of *Salmonella enterica* are located on the chromosomal *Salmonella* pathogenicity islands (SPI), which are large mobile genetic elements (MGE), capable of acquiring genes as gene cassettes [42,43,44]. There are 24 *Salmonella* pathogenicity islands that have been identified [45,46]; however, it is worth noting that not all these SPIs have been scientifically validated to contribute to phenotypic virulence [47,48,49], with SPI-1, SPI-2, SPI-3, SPI-4, and SPI-5 being abundant in most *Salmonella* serovars [45,50,51,52]. *SivH* is a pathogenicity determinant implicated in the intracellular survival of *Salmonella*. The high prevalence of *SivH* genes can be attributed to their association with islands that are specific to *Salmonella* capable of infecting warm-blooded animals [53].

South Africa has legislation in place that makes it a contravention to obtain prescribed antimicrobial medicines without a prescription; however, the Farm Feeds, Agriculture Remedies, Fertilizers, and Stock Remedies Act (Act No. 36, 1947 [54]) permits the acquisition of listed stock remedies for livestock treatment without a prescription [55]. In low- and middle-income countries (LMICs) such as South Africa, there is little information regarding the occurrence and distribution of NTS on chicken carcasses during slaughtering process and its antimicrobial resistance patterns [11,56,57]. Therefore, the aim of this study was to evaluate the presence of *Salmonella* along the slaughter process in low- and high-throughput poultry abattoirs in South Africa and to determine their characteristics.

## 2. Materials and Methods

### 2.1. Ethical Approval

Ethical approval for this study was obtained from University of Zululand research ethics committee with reference number UZREC 171110-030 PGM 2022/61. This study was a part of an umbrella project for Prof. Evelyn Madoroba, with ethics approval reference UZREC 171110-030 Dept 2022/11.

### 2.2. Study Design, Study Site, and Sample Collection

This study was conducted on abattoirs that were randomly selected based on the 2019 European Food Safety Authority (EFSA) and European Centre for Disease Prevention and Control (ECDPC) [58] reports for monitoring foodborne pathogens. This study used the sample size determination equation for cross-sectional analysis to determine the appropriate sample size [59,60].

$$\mathrm{Sample size}=\frac{{\left(Z\alpha \right)}^{2\;}*p(1-p)\;}{d^{2}}$$

Zα is standard normal variant (5% type 1 error, i.e., 1, 96), *p* is the estimated prevalence from previous studies, and *d* is the unconditional error (*p* < 0.05).

$$=\frac{1.96^{2}*0.50\left(1-0.50\right)}{0.05^{2}}$$

= 384 poultry samples

Assuming a prevalence of 50%, the required minimum sample size is 384. To increase the robustness of the conclusions, the poultry sample size for this study was 500.

In South Africa, abattoirs are divided into three categories: rural, high-throughput (HT), and low-throughput (LT) [19]. Between July and September 2022, 500 chicken rinsates were sampled from two low-throughput and one high-throughput abattoir. Rinsate samples were collected randomly at three processing stages (scalding, evisceration, final water). This was performed to have good representation of *Salmonella* prevalence at different critical stages in KZN abattoirs. There was no particular stratification. Scalding was conducted at 50–55 °C in order to loosen the chicken feathers and to ensure that the carcasses are not over scalded [19]. Prior to evisceration, the chicken carcasses were washed according to the provisions in South Africa’s Foodstuffs, Cosmetics, and Disinfectants Act, 1972 (Act No. 54 of 1972). Evisceration was conducted in a hanging position on the evisceration line. After evisceration, the chicken carcasses were washed with cold water according to the provisions of South Africa’s Foodstuffs, Cosmetics, and Disinfectants Ad, 1972 (Act No. 54 of 1972). The samples for scalding water were collected immediately from a channel. Evisceration samples were taken from water obtained from washing individual chicken carcasses. Chicken rinsates were collected by placing the sterile containers below the carcasses as they were washed on a line.

**Figure 1:**
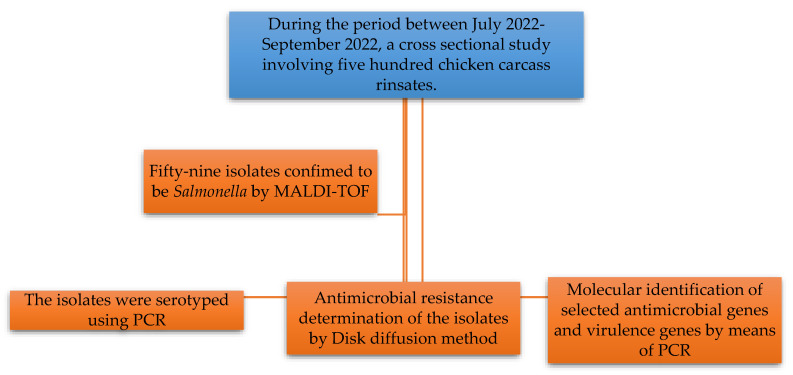
Schematic representation of the workflow used for this study.

Chicken carcass rinsates were aseptically placed in sterile Stomacher bags and delivered to the University of Zululand main campus in Kwa-Dlangezwa on ice for microbiological testing and analysis within 12 h. Figure 1 illustrates the schematic representation of the workflow used for this study.

### 2.3. Microbiological Analysis and Cryopreservation of Isolates

#### Isolation and Identification of *Salmonella*

Detection and phenotypic confirmation of *Salmonella* was performed according to the ISO-6579-1:2017/Amd 1:2020 International Organization for Standardization (ISO-6579-1:2017/Amd 1:2020 [61] protocol with slight modification. Briefly, the method involved pre-enrichment whereby 10 mL of each sample was measured and aseptically transferred into 90 mL of the pre-enrichment broth, buffered peptone water (BPW) (Oxoid Ltd., Hampshire, UK), and incubated at 37 °C for 18–24 h. Selective enrichment was performed after incubation, whereby 0.1 mL and 1 mL aliquots of the samples were inoculated into 10 mL Rappaport–Vassiliadis soy broth (RVS) (Oxoid, UK) and Muller–Kauffmann tetrathionate-novobiocin broth (MKTTn), respectively (Oxoid, UK), followed by incubation at 37 ± 2 °C and 42 ± 2 °C for 24 ± 2 h, respectively. Loopfuls of the samples from the selective broths (MKTTn and RVS) were streaked onto xylose lysine deoxycholate (XLD) agar (Oxoid, UK) and Bismuth sulphide agar (BSA) (Oxoid, UK), followed by incubation at 37 ± 2 °C for 24 ± 2 h. Colonies that were pink and black centred on XLD and had black metallic sheen on BSA were considered presumptive *Salmonella*.

Presumptive isolates were purified on XLD and on nutrient agar (Oxoid, UK), followed by incubation at 37 ± 2 °C for 24 ± 2 h. All presumptive *Salmonella* were cryopreserved in stock cultures of nutrient broth supplemented with sterile glycerol to a final concentration of 50%, followed by storage at −80 °C until required. The presumptive isolates were also preserved in 1 mL of 10% skimmed milk and stored at −80 °C.

### 2.4. Salmonella Identification Using MALDI-TOF MS

All preserved *Salmonella* isolates were revived and sub-cultured on XLD to ensure purity. The revived presumptive *Salmonella* isolates were subjected to MALDI-TOF MS for species confirmation according to the manufacturer’s instruction [62,63].

### 2.5. PCR Serotyping of Salmonella Isolates

The predominant *Salmonella* serovars in poultry are usually *Salmonella enterica* serovar Enteritidis and *Salmonella enterica* serovar Typhimurium in Africa [6]; hence, this study focused on these two serovars. Conventional PCR was undertaken to screen for *S.* Enteritidis and *S*. Typhimurium. The forward and reverse primer sequences for each gene are shown in Table 1.

A 20 µL master mix consisting of 10 µL NEB OneTaq 2X MasterMix with Standard Buffer (Inqaba Biotec, Pretoria, South Africa; Catalogue No. M0482S), 1 µL of genomic DNA (10–30 ng/μL), 1 µL forward primer (10 μM), and 1 µL reverse primer (10 μM) was used. The volume was made up to 20 µL with nuclease-free water (Inqaba Biotec; Catalogue No. E476). Thermocycling of the PCR mixture was conducted under the following conditions: initial denaturation at 94 °C for 5 min; 35 cycles of denaturation at 94 °C for 30 s, annealing at 50 °C for 30 s; extension at 68 °C for 1 min; final elongation at 68 °C for 10 min; and the amplicons were kept at 4 °C for further analysis, as previously described [27].

### 2.6. Agarose Gel Electrophoresis of PCR Amplicons

The PCR amplicons were electrophoresed in a 1.5% agarose gel (CSL-AG100, Cleaver Scientific Ltd, Rugby, UK) and stained with ethidium bromide at 90 V for approximately 30 min. A 100 bp NEB Fast Ladder (N3238; (Inqaba Biotec, Pretoria, South Africa)) was used to estimate the size of the amplicons. The results were viewed and documented using the VILBER (E-Box; Vilber, Eberhardzell, Germany) gel documentation system.

### 2.7. Antimicrobial Susceptibility

The Kirby–Bauer disk diffusion method [66] was used to determine the antimicrobial susceptibility of confirmed *Salmonella* isolates. The strains were tested against commonly used antibiotics in poultry farming [58]: ampicillin (10 µg), chloramphenicol (30 µg), trimethoprim–sulfamethoxazole, oxytetracycline (10 µg), cefotaxime, gentamicin (10 µg), ciprofloxacin, and kanamycin (30 µg) (Davies Diagnostic, Randburg, South Africa). Briefly, four to five colonies were inoculated into 5 mL of sterile physiological saline solution, and the turbidity of the suspension was adjusted to match the 0.5 McFarland standard. Subsequently, the standardized *Salmonella* bacterial suspensions were swabbed onto Mueller–Hinton agar plates (Thermo-Fisher, Waltham, MA, USA) in three directions, rotating the plate by approximately 60° angles between each swabbing to ensure uniform distribution. Afterwards, the inoculated medium was allowed to dry for about five to ten minutes, and antibiotic disks were applied on the Mueller Hinton agar. Following a 16 h incubation period at 37 °C ± 2, the resulting zones of inhibition were measured in millimetres (mm). The obtained results were then classified as susceptible (S), intermediate (I), or resistant in accordance with the guidelines set by the Clinical Laboratory Standards Institute (CLSI) [66].

### 2.8. Evaluation of Antimicrobial Resistant Genes Using PCR

#### DNA Extraction

The conventional cell-lysis (boiling) method was used as previously described [25]. Briefly, the *Salmonella* isolates were resuscitated on nutrient agar and incubated at 37 ± 2 °C for 24 ± 2 h. A loopful of the culture medium was inoculated into an Eppendorf tube containing 1 mL of sterile distilled water. The inoculum was thoroughly mixed and then placed in a heating block at 99 °C for 10 min. It was then centrifuged at 13,000 rpm for 5 min. The pellet was discarded, and the DNA-containing supernatant was transferred to a sterile tube for PCR amplification of antimicrobial genes and virulence genes.

Multiplex PCR was used for the screening of selected antimicrobial resistance genes of *Salmonella* strains. Twelve antimicrobial resistance genes previously described by Lauteri and co-workers [67] were screened with slight modifications: *bla_TEM_* and *bla_PSE_* for beta-lactam resistance; *aadA2*, *aac*(*3)IV*, and *aadB* for aminoglycoside resistance; *catA1* for chloramphenicol resistance; *tetA*, *tetB*, and *tetC* for tetracycline resistance; *dfrA1* and *dfrA14* for trimethoprim resistance. The forward and reverse primer sequences for each gene is shown in Table 2.

The 25 µL PCR mixtures consisted of 12.5 μL NEB OneTaq 2X MasterMix with standard buffer (Inqaba Biotec, Pretoria, South Africa; Catalogue No. M0482S), 5 µL Genomic DNA (15–150 ng/μL), 1 μL of each of the forward and reverse primer, and the volume was topped up with molecular-grade water (Inqaba Biotec, Pretoria, South Africa; Catalogue No. E476). The thermocycling conditions consisted of 94 °C for 5 min of initial denaturation, 35 cycles at 94 °C for 30 s of denaturation, 50 °C for 30 s, 68 °C for 1 min of elongation, and 68 °C for 10 min of final extension. Agarose gel electrophoresis of PCR amplicons was conducted as described in Section 2.6.

### 2.9. Evaluation of Salmonella spp. Virulence Genes

The virulence genes screened in this study were previously described by Siddiky et al. (2021) [72] and included invasion proteinA (*invA*), aggregative fimbriae A (*agf*A), long polar fimbrial A (*lpf*A), and intimin-like inverse autotransporter protein H (*Siv*H). The forward and reverse primer sequences for each gene is shown in Table 3.

A 25 µL PCR master mixture was prepared consisting of 12.5 μL NEB OneTaq 2X MasterMix with standard buffer (Inqaba Biotec; Catalogue No. M0482S), 5 µL crude DNA (50–150 ng/μL), 5.5 µL molecular-grade water (Inqaba Biotec; Catalogue No. E476), and 1 μL of each of the forward and reverse primers. For amplification, the mixtures underwent thermocycling under the following conditions: for initial denaturation, 94 °C for 5 min; followed by 35 cycles of denaturation at 94 °C for 30 s; annealing at 50 °C for 30 s; elongation at 68 °C for 1 min; and final extension was at 68 °C for 10 min.

### 2.10. Agarose Gel Electrophoresis of PCR Amplicons

The PCR amplicons were electrophoresed on 1.5% agarose gels stained with ethidium bromide at 90 V for approximately 30 min. A 100 bp NEB Fast Ladder (N3238; (Inqaba Biotec, Pretoria, South Africa)) was used to estimate the size of the amplicon. The results were viewed and documented using VILBER (E-Box; Vilber, Eberhardzell, Germany) gel documentation.

### 2.11. Quality Control

To ensure the accuracy and validity of the results obtained in this study, reference strains *Salmonella* Typhimurium ATCC14028 and *Escherichia coli* ATCC 25922 were included in all experiments, as positive and negative controls, respectively [25]. Previous positive isolates obtained from Prof. Madoroba’s research group [23,27] were used as positive controls for virulence genes. Standard method validation approaches for all methods were undertaken.

### 2.12. Statistical Analysis

Confidence intervals (CI) were calculated using binomial distribution in Excel. The results of the prevalence of *Salmonella* spp. were compared between abattoirs using the chi square test. A *p*-value < 0.05 was considered statistically significant.

## 3. Results

### 3.1. MALDI-TOF Identification

From the eighty-one presumptive *Salmonella* isolates, fifty-nine *Salmonella* isolates were confirmed to be *Salmonella*. As a result, the overall prevalence of *Salmonella* in KZN abattoirs was 11.8% (59/500, CI: 9.5–15). *Salmonella* prevalence was reported to be 39.58% (19/48) in LT abattoir A rinsates, and 17.32% (40/231) in HT abattoir rinsates, with no *Salmonella* identified on chicken carcass rinsates in LT abattoir B. *Salmonella* occurrence was the highest during evisceration stage (21.01%), followed by final washing (10.28%), and no *Salmonella* was isolated during scalding. Table 4 summarizes the prevalence of *Salmonella* isolates from various processing stages in KZN abattoirs.

### 3.2. PCR Serotyping

**Figure 2:**
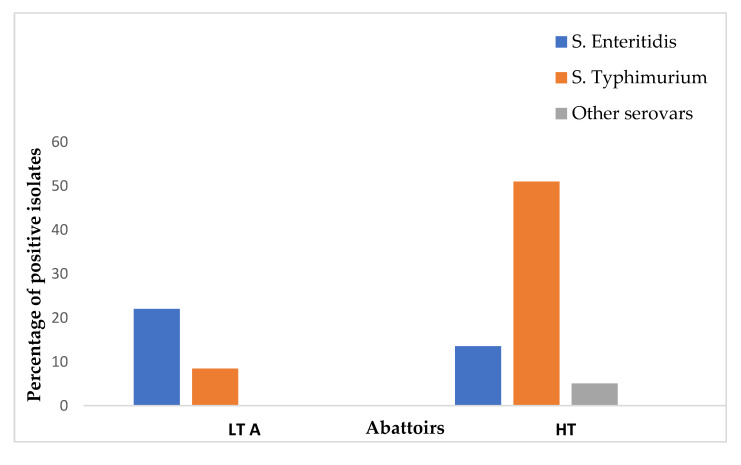
Distribution of *Salmonella* serovars of chicken carcass rinsates from low-throughput and high-throughput abattoirs in KZN.

**Figure 3:**
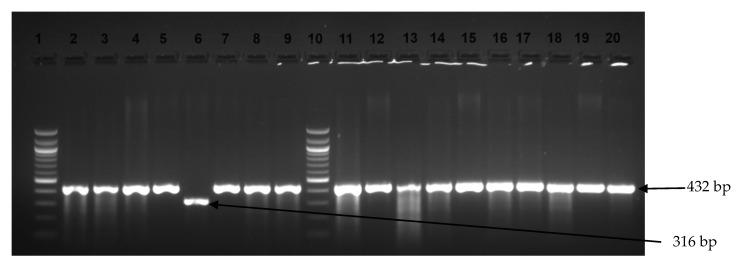
Image showing examples of *Salmonella* Typhimurium and *S*. Enteritidis amplicons observed on an agarose gel. Lanes 1 and 10 show the 100 bp DNA ladder, while lanes 2–5, 7–9, and 11–20 display positive amplification for *S*. Typhimurium (432 bp). Lane 6 shows *S*. Enteritidis amplicons (316 bp).

**Figure 4:**
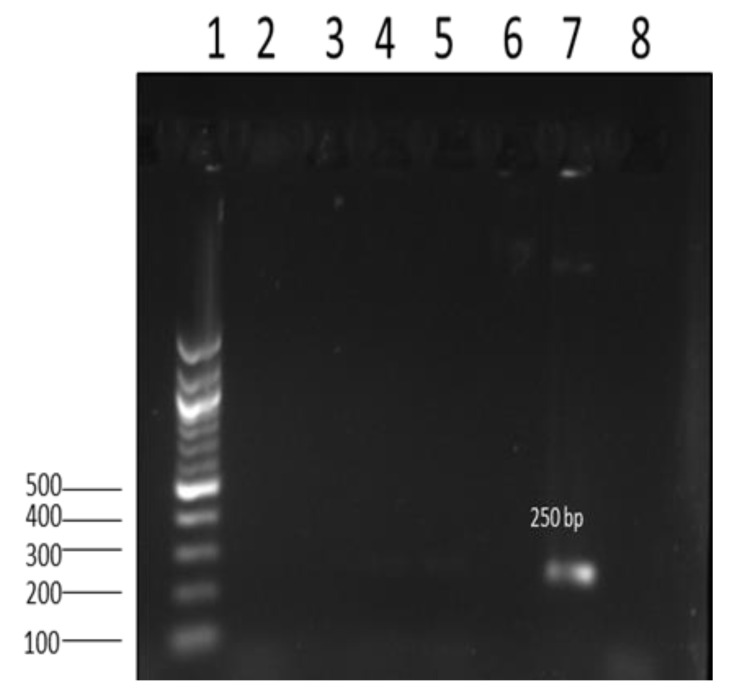
Image showing amplicon of antimicrobial resistance gene *aad*2 (250 bp) from *Salmonella* isolates. Lane 1: 100 bp DNA ladder; lane 2–6; negative results for the *aad*2 gene; lane 7: positive 250 bp *aad*2 gene; lane 8: nuclease-free water (negative control).

Of the 59 *Salmonella* isolates, 35 were *S.* Typhimurium (59.3%, 95% CI 45.7–72), 21 were *S*. Enteritidis (35.6%, 95% CI 23.6–49), and only 3 were neither *S*. Typhimurium nor *S*. Enteritidis. The prevalences of *S.* Enteritidis and *S*. Typhimurium were found to be 22.03% (13/59) and 8.47% (5/59) in LT A, respectively. Similarly, the prevalences of *S*. Enteritidis, *S*. Typhimurium, and other serovars were represented as 13.56% (8/59), 51% (30/59), and 5.08% (3/59) in the HT abattoir, respectively (Figure 2). All genes that were screened in this study are illustrated in Figure 3 and Figure 4.

### 3.3. Antimicrobial Susceptibility Testing

We found a low presence of antimicrobial resistance. All of the isolates from the LT slaughterhouse were susceptible. However, in the HT abattoir, a multi-resistant *S.* Enteritidis strain with the phenotypic-pattern ampicillin, oxytetracycline, cefotaxime, and trimethoprim/sulphonamide, as well as two *S.* Typhimurium strains, were resistant to oxytetracycline and ampicillin, while the remaining isolates were susceptible to all antibiotics.

### 3.4. Evaluation of Virulence Genes Using PCR

Table 5 shows the distribution of the screened virulence genes according to their serotypes and origin. All *S.* Enteriditis were positive for *invA*, while two *S.* Typhimurium strains were negative. Other virulence genes tested were present in 40-to-100% (*siv*H in *S.* Typhimurium) of the strains. The strains with an unknown serotype were positive for nearly all virulence genes except *agf*A.

### 3.5. Evaluation of Antimicrobial Resistance Genes Using PCR

Three of the ten AMR genes tested were found in *Salmonella* isolates recovered from the two (LT A and HT) abattoirs. Overall, the most common AMR genes were *tet*A 54.2% (32/59), *aad*A2 (aminoglycosides) at 3.4%, and *bla*_TEM_ (beta-lactam) at 1.7%. All of the isolates tested negative for the following AMR genes: *aac(3)IV*, *aad*B (aminoglycosides), *bla*_PSE_ (beta-lactam), *tet*B, *tet*C (*Tet*racycline), *cat*A1 (phenicol), and *dfr*A1 (trimethoprim) (Table 6).

## 4. Discussion

Non-typhoidal *Salmonella* is a significant zoonotic pathogen posing a threat to human health [73]. *Salmonella* spp. was detected in 11.8% of the 500 rinsates from chicken carcasses collected during this study. The presence of NTS in chicken rinsates in this study is concerning because it can lead to the cross-contamination of poultry and the environment through contaminated water sources. These contaminated sources may lead to non-typhoidal salmonellosis, which is listed in the South African National Health Act, 2003 (Act No. 61 of 2003), under Category 3 of notifiable medical conditions. Furthermore, *S*. Enteritidis, which was one of the predominant serovars in this study, is listed in South Africa as a controlled disease on the List of Controlled and Notifiable Animal Diseases in Terms of the Animal Diseases Act, 1984 (Act No. 35 of 1984). Therefore, routine surveillance and strict hygiene should be practiced during the slaughter of chickens as abattoirs may serve as potential sources of *Salmonella* contamination in humans.

The rate of *Salmonella* occurrence in this study was found to be higher compared to a previous study in KwaZulu-Natal, which reported a prevalence 0.6% (1/162) *Salmonella* from carcass rinsates at an abattoir [11]. The difference in prevalence between our study and that of Ramtahal et al. (2022) [11] could be due to a variety of differences, including, but not limited to, the sample size (500 samples from this study compared to 162), the source of the samples, and the sampling strategy.

We found a higher prevalence in HT abattoirs, followed by the LT A abattoir, with no *Salmonella* occurrence in the LT B abattoir. In high-throughput slaughterhouses, contamination risks can be particularly high due to the large number of animals processed, the use of automation, and the high throughput rate. The speed of processing and high volume of animals can make it difficult to maintain adequate hygiene practices and to monitor for contamination effectively. In the low-throughput abattoir A, *Salmonella* contamination could be a result of a lack of resources, inadequate facilities, and limited regulatory oversight. No *Salmonella* was detected in the low-throughput B abattoir, which could suggest that good hygiene practices were observed during the processing of the flocks or that the chickens were not contaminated during production at the farm or during transportation to the abattoir. Further studies are necessary to verify the data as only a few slaughterhouses were included in this study, and there may be differences in operationality between the slaughterhouses. Studies accounting for these differences would be expected to determine better practices.

The reduction of *Salmonella* at the final carcass-washing stage is a clear indicator that interventions at the point of carcass washing can reduce the level of the bacterial contamination of carcasses to below detection. No *Salmonella* was isolated from the scalding water, which is perhaps an indication of the implementation of good hygiene measures in slaughtering and processing [25]. The temperature of the scalding water could be a hurdle that reduces the bacterial contamination of chicken carcasses.

The predominant *Salmonella* serovars identified in this study were *S.* Enteritidis and *S.* Typhimurium. These serovars have been shown to be predominant in different studies and have been associated with most cases of human salmonellosis. In South Africa, a retrospective study involving the incidence of *Salmonella* from different animal species from 1996 to 2006 revealed that out of the 183 serovars, *S*. Typhimurium was predominant, followed by *S*. Dublin and *S*. Enteritidis [74]. A study that was undertaken in Iran to determine *Salmonella* species in poultry markets revealed *S*. Typhimurium as the dominant and only serovar that was recovered [75]. A systematic review and meta-analysis in South Africa that used pooled prevalence to assess the “One Health” perspective of *Salmonella* serovars found *S*. Typhimurium and *S*. Enteritidis to constitute a relatively large proportion [9]. In Saudi Arabia, the prevalences of *S.* Enteritidis and *S.* Typhimurium associated with human infections in Riyadh were 40% and 13.5%, respectively [76].

While most of the *Salmonella* isolates from this study were susceptible to the antibiotics tested, one *S*. Enteritidis strain showed multi-drug resistance (MDR). Previously in KZN, all of the *Salmonella* isolates from wastewater, and 72.4% of carcass rinsates were susceptible to all of the tested antibiotics [11], while a study covering South Africa [56] found a high prevalence of resistance to tetracycline (93%), kanamycin (74%), trimethoprim–sulfamethoxazole (50%), gentamicin (48%), ampicillin (47%), and chloramphenicol (31%). The MDR *S*. Enteritidis strain from this study was also resistant to cefotaxime. The presence of resistance against third-generation cephalosporins is a worrisome observation as these strains may be selected by most beta-lactam antibiotics, as well as through co-resistance selection. Cefotaxime resistance is highly concerning as this antimicrobial is considered a potent treatment for septicaemic salmonellosis in adult humans. The emergence of resistant isolates highlights the urgent need for regular surveillance to prevent the spread of resistant strains.

Resistance to tetracyclines was low at the phenotypic level, but about half of the strains showed the presence of *tetA* genes. These are referred to as “silent” or “cryptic” antimicrobial resistance genes [77,78,79,80]. The reasons for this discrepancy in phenotypic and genotypic occurrence remain under investigation. Nevertheless, the presence of *tetA* genes does not always imply that the bacteria show phenotypic resistance [77,78,79,80]. However, “silent” antimicrobial resistance genes may have public health consequences because when the conditions are suitable, the “silent” genes may be expressed and regain resistance, which may lead to the failure of therapeutic agents [77]. Some of the resistance genes that were observed from this study were previously identified from other similar studies albeit from different sampling sites in South Africa [55,56]. It is important to use a holistic multidisciplinary “One Health” approach that takes into consideration the health of the animals, the environment, and the health of human beings to reduce microbial contamination and curb the spread of antimicrobial resistance [81,82].

Virulence genes play a crucial role in the pathogen’s ability to survive and multiply within the host cell. In this study, *inv*A was the most prevalent virulence gene. This gene is located inside the *Salmonella* pathogenicity island-1 (SPI-1) [83] and is associated with the Type III Secretion System (T3SS), which aids in host cell invasion. The *inv*A gene also serves as a biomarker for NTS *Salmonella* as it is present in nearly all *Salmonella enterica* subspecies *enterica* [83]. In this study, two isolates did not carry the *invA* gene. It has been reported that *inv*A gene-negative *Salmonella* strains are rare and have been observed in few studies [23,84]. In South Africa, Naidoo et al. (2022) observed *invA*-negative *Salmonella enterica* Heidelberg from beef tripe [23]. As the *invA* gene plays a role in the invasion of epithelial cells during infection, *Salmonella* strains that are negative for the *invA* gene may invade epithelial cells using other mechanisms [85].

The *agf*A and *IpfA* genes are crucial to the infection process as they encode fimbriae, which play a vital role in the attachment process [72]. *agf*A is also involved in the process of biofilm formation, and the presence of this gene in *Salmonella* represents a risk of persistence on slaughterhouse equipment and other surfaces [86]. Other studies have also documented the occurrence of the *agf*A gene in *S.* Enteriditis and *S.* Typhimurium [23,27]. *sivH* (also known as *sinH*), an intimin-like inverse autotransporter protein, is involved in the internal colonization and persistence of *Salmonella* [72], indicating that most of the strains in this study are capable of maintaining their presence in the poultry population.

## 5. Conclusions

In this study, we determined the occurrence, serotypes, phenotypical antimicrobial resistance profiles, selected antibiotic resistance genes, and virulence factors of NTS in chicken rinsates from abattoirs located in selected areas of KwaZulu-Natal province, South Africa. Our findings show that chicken meat serves as a potential carrier of zoonotic *Salmonella* spp. With the exception of the identified *S*. Enteritidis MDR isolate, low antimicrobial resistance was observed in this study. However, despite the low resistance to tetracyclines at the phenotypic level, almost half of the strains showed the presence of *tetA* genes, which may be due to “silent” or “cryptic” antimicrobial resistance genes. The *Salmonella* harbored diverse virulence genes, which is important for pathogenicity and infectivity. Therefore, higher standards of basic hygiene, hygiene awareness, and proficient food management abilities in the abattoirs are recommended to reduce cross-contamination. Cross contamination was more pronounced in HT slaughterhouses and slaughter processes; therefore, hygiene and hazard analysis and critical control points should be reanalyzed and improved to reduce contamination levels. Further molecular epidemiology studies are necessary, including detailed characterization of NTS strains with whole-genome sequencing, which is critical for the prevention, reduction, and elimination of contaminants in the food chain.

## Figures and Tables

**Table 1:** Primer sequences used to screen for *S*. Enteritidis and *S*. Typhimurium using PCR.

Target Genes	Sequence (5′ to 3′)	Size (bp)
Insertion Element (IE-1)	F-AGTGCCATACTTTTAATGAC R-ACTATGTCGATACGGTGGG	316
F Flic-C	F-CCCGCTTACAGGTGGACTAC R-AGCGGGTTTTCGGTGGTTGT	432

References: [64,65].

**Table 2:** Primer sequences used to screen for each of the thirteen antimicrobial resistance genes in *Salmonella* spp. using multiplex PCR *.

Target Gene	Sequence (5′ to 3′)	Amplicon Size (bp)
*aadA2*	F-CGGTGACCATCGAAATTTCG R-CTATAGCGCGGAGCGTCTCGC	250
*aadB*	F-GAGGAGTTGGACTATGGATT R-CGGATGCAGGAAGATCAA	208
*aac*(*3)IV*	F-TGCTGGTCCACAGCTCCTTC F-TGCTGGTCCACAGCTCCTTC	653
*bla_TEM_*	F-CCGTGTCGCCCTTATTCCC R-GCCTGACTCCCCGTCGTG	780
*bla^PSE^*	F-CGCTTCCCGTTAACAAGTAC R-CTGGTTCATTTCAGATAGCG	465
*catA1*	F-GGCATTTCAGTCAGTTG R-CATTAAGCATTCTGCCG	551
*tetA*	F-GTAATTCTGAGCACTGT R-CCTGGACAACATTGCTT	954
*tetB*	F-ACGTTACTCGATGCCAT R-AGCACTTGTCTCCTGTT	1170
*tetC*	F-AACAATGCGCTCATCGT R-GGAGGCAGACAAGGTAT	1138
*dfrA1*	F-GTGAAACTATCACTAATGG R-TTAACCCTTTTGCCAGATTT	474
*dfrB*	F-GATCACGTGCGCAAGAAATC R-AAGCGCAGCCACAGGATAAAT	141

* References: adopted from [67,68,69,70,71].

**Table 3:** Primer sequences to screen for each of the four virulence genes in *Salmonella* spp. using PCR *.

Target Gene	Sequence (5′ to 3′)	Amplicon Size (bp)
*inv*A	F-GTGAAATTATCGCCACGTTCGGGCAA R-TCATCGCACCGTCAAAGGAACC	284
*agf*A	F-TCCACAATGGGGCGGCGGCG R-CCTGACGCACCATTACGCTG	350
*Ipf*A	F-CTTTCGCTGCTGAATCTGGT R-CAGTGTTAACAGAAACCAGT	250
*Siv*H	F-GTATGCGAACAAGCGTAACAC R-CAGAATGCGAATCCTTCGCAC	763

* Reference: [72].

**Table 4:** Prevalence of *Salmonella* in different chicken processing stages classified according to the abattoir category.

Processing Stages	Percentage (%) of *Salmonella* Positive Samples (n)	CI (%)	Abattoir		
			**LT A**	**LT B**	**HT**
Scalding water	0(0/80)	0	-	0(0/80)	-
Evisceration	21.01 (32/138)	16.4–31	-	0(0/23)	27.83 (32/115)
Final water	10.28 (27/282)	6.4–14	39.58 (19/48)	0(0/118)	6.9 (8/116)
Total	11.8 (59/500)	9.5–15	39.58 (19/48)	0(0/221)	17.32 (40/231)

LT A refers to the low-throughput abattoir A; LT B refers to the low-throughput abattoir B; HT refers to high-throughput abattoir; - implies that there were no samples for the specified categories.

**Table 5:** Distribution of virulence genes in *Salmonella enterica* serotypes from chicken carcasses in LT A and HT abattoirs.

Sites	Serovars	*inv*A (%)	*agf*A (%)	*Ipf*A (%)	*siv*H (%)
LT A	*S*. Enteritidis	100 (13/13)	76.92 (10/13)	76.92 (10/13)	84.62 (11/13)
	*S.* Typhimurium	100 (5/5)	60 (3/5)	40 (2/5)	100 (5/5)
HT	*S*. Enteritidis	100 (8/8)	87.5 (7/8)	75 (6/8)	87.5 (7/8)
	*S.* Typhimurium	93.33 (28/30)	86.7 (26/30)	73.33 (22/30)	80 (24/30)
	Non-serotyped	100 (3/3)	66.67 (2/3)	100 (3/3)	100 (3/3)

LT A—low-throughput A; HT—high-throughput.

**Table 6:** Frequency of AMR genes in *S*. Typhimurium, *S*. Enteritidis, and other serotypes in different chicken abattoirs.

Site	Serovars	*aad*A2 %	*aac*(3)IV %	*aadB* %	*bla_TEM_* %	*bla_PSE_* %	*tet*A %	*tet*B %	*tet*C %	*catA1* %	*dfrA1* %
LT A	*S.* Typhimurium	0 (0)	0 (0)	0 (0)	0 (0)	0 (0)	75 (3/4)	0(0)	0 (0)	0 (0)	0 (0)
	*S*. Enteritidis	0 (0)	0 (0)	0 (0)	7.7 (1/13)	0 (0)	84.6 (11/13)	0 (0)	0 (0)	0 (0)	0 (0)
HT	*S*. Typhimurium	6.5 (2/31)	0 (0)	0 (0)	0 (0)	0 (0)	48.4 (15/31)	0 (0)	0 (0)	0 (0)	0 (0)
	*S*. Enteritidis	0 (0)	0 (0)	0 (0)	0 (0)	0 (0)	14.3 (1/7)	0 (0)	0 (0)	0 (0)	0 (0)
	Non-serotyped	0 (0)	0 (0)	0 (0)	0 (0)	0 (0)	66.7 (2/3)	0 (0)	0 (0)	0 (0)	0 (0)
	Total	3.4 (2/59)	0 (0)	0 (0)	1.7 (1/59)	0 (0)	54.3 (32/59)	0 (0)	0 (0)	0 (0)	0 (0)
	*p-*value	0.4015	-	-	7.140	-	0.0215 *	-	-	-	-

* significant (*p* < 0.05). LT A—low-throughput A; HT—high-throughput.

## Data Availability

The original contributions presented in this study are included in the article. Further inquiries can be directed to the corresponding author.
